# Genomic investigation of *Lactococcus formosensis*, *Lactococcus garvieae*, and *Lactococcus petauri* reveals differences in species distribution by human and animal sources

**DOI:** 10.1128/spectrum.00541-24

**Published:** 2024-04-30

**Authors:** You-Xiang Chan, Huiluo Cao, Shuo Jiang, Xin Li, Ka-Kin Fung, Chung-Ho Lee, Siddharth Sridhar, Jonathan Hon-Kwan Chen, Pak-Leung Ho

**Affiliations:** 1 Department of Microbiology, University of Hong Kong, Hong Kong, China; 2 Carol Yu Centre for Infection, University of Hong Kong, Hong Kong, China; 3 Department of Microbiology, Queen Mary Hospital, Hospital Authority, Hong Kong, China; 4 Department of Clinical Pathology, Pamela Youde Nethersole Eastern Hospital, Hospital Authority, Hong Kong, China; 5 Department of Clinical Pathology, Kwong Wah Hospital, Hospital Authority, Hong Kong, China; Taichung Veterans General Hospital, Taichung, Taiwan, China

**Keywords:** zoonotic infections, genomics, virulence determinants, molecular epidemiology

## Abstract

*Lactococcus garvieae* is a fish pathogen that can cause diseases in humans and cows. Two genetically related species, *Lactococcus formosensis* and *Lactococcus petauri*, may be misidentified as *L. garvieae*. It is unclear if these species differ in host specificity and virulence genes. This study analyzed the genomes of 120 *L*. *petauri*, 53 *L*. *formosensis*, and 39 *L*. *garvieae* isolates from various sources. The genetic diversity and virulence gene content of these isolates were compared. The results showed that 77 isolates previously reported as *L. garvieae* were actually *L. formosensis* or *L. petauri*. The distribution of the three species varied across different collection sources, with *L. petauri* being predominant in human infections, human fecal sources, and rainbow trout, while *L. formosensis* was more common in bovine isolates. The genetic diversity of isolates within each species was high and similar. Using a genomic clustering method, *L. petauri*, *L. formosensis*, and *L. garvieae* were divided into 45, 22, and 13 clusters, respectively. Most rainbow trout and human isolates of *L. petauri* belonged to different clusters, while *L. formosensis* isolates from bovine and human sources were also segregated into separate clusters. In *L. garvieae*, most human isolates were grouped into three clusters that also included isolates from food or other sources. Non-metric multidimensional scaling ordination revealed the differential association of 15 virulence genes, including 14 adherence genes and a bile salt hydrolase gene, with bacterial species and certain collection sources. In conclusion, this work provides evidence of host specificity among the three species.

**IMPORTANCE:**

*Lactococcus formosensis* and *Lactococcus petauri* are two newly discovered bacteria, which are closely related to *Lactococcus* garvieae, a pathogen that affects farmed rainbow trout, as well as causes cow mastitis and human infections. It is unclear whether the three bacteria differ in their host preference and the presence of genes that contribute to the development of disease. This study shows that *L. formosensis* and *L. petauri* were commonly misidentified as *L. garvieae*. The three bacteria showed different distribution patterns across various sources. *L. petauri* was predominantly found in human infections and rainbow trout, while *L. formosensis* was more commonly detected in cow mastitis. Fifteen genes displayed a differential distribution among the three bacteria from certain sources, indicating a genetic basis for the observed host preference. This work indicates the importance of differentiating the three bacteria in diagnostic laboratories for surveillance and outbreak investigation purposes.

## INTRODUCTION

*Lactococcus garvieae* is a Gram-positive bacterium that was first isolated from bovines with mastitis and previously described as *Streptococcus garvieae* (1). In 1985, it was reclassified into the new genus *Lactococcus* (2). The bacterium causes mastitis in ruminants and lactococcosis in fish, which are of special relevance in animal husbandry and farmed fish. This bacterium has been isolated from various marine and fresh fish species, prawns, and wild marine animals (2, 3). It has also been found in pigs with pneumonia, and cat and dog tonsils. The organism has been isolated from fish products, raw milk, goat cheese, meat products, vegetables, and cereals (2, 3). The bacterium has low virulence in humans and is considered to be an opportunistic pathogen (4, 5). Cases of *L. garvieae* causing bacteremia, infective endocarditis, peritonitis, liver abscess, osteomyelitis, spondylitis, urinary tract infection, and meningitis have been described (2). Human infection with *L. garvieae* has been associated with the consumption of raw fish, seafood, and unpasteurized milk; occupational fishery exposure; and seasonal outbreaks of infections in aquaculture (2, 6, 7).

In the past decade, reports of human infections by *L. garvieae* appear to be increasing and may be related to the use of improved bacterial identification methods and higher awareness among clinicians (4, 5). However, its accurate identification is hampered by its close taxonomic relationship with *Lactococcus formosensis* and *Lactococcus petauri*, which were designated as novel species in 2014 and 2017, respectively (8, 9). These three *Lactococcus* species have very similar phenotypic and biochemical properties, making conventional methods inadequate for their identification. Currently, *L. formosensis* and *L. petauri* are not included in the databases of the matrix-assisted laser desorption/ionization–time-of-flight mass spectrometry(MALDI-TOF MS)-based identification systems, and these organisms would likely be misidentified as *L. garvieae* (10, 11). Methods based on the 16S rRNA gene are also widely used in medical microbiology for bacterial identification. However, recent literatures have confirmed that 16S rRNA-based approaches are not adequate for the identification of members of the *Lactococcus* genus, as the 16S rRNA sequences of these three *Lactococcus* species are highly similar (12, 13).

Although lactococcosis in fish is historically attributed solely to *L. garvieae*, recent investigations have shown that the disease can also be caused by *L. petauri* and *L. formosensis*. The first outbreak attributed to *L. petauri* infection occurred in a Greek facility in 2007, involving farmed rainbow trout (14, 15). The causative organism was initially identified as *L. garvieae* by biochemical methods and PCR (15). In a retrospective genome analysis, the etiological agent was reclassified as *L. petauri* (14). In 2020, several large outbreaks of lactococcosis occurred in cultured rainbow trout in Southern California resulting in the cull of over 3.2 million fish due to ineffective control measures (16). The organisms involved in these outbreaks were initially identified as *L. garvieae*, but whole-genome sequencing (WGS) later identified the causative agent as *L. petauri* (16, 17). These developments suggest that human infections, carriage, or food contamination by *L. petauri* and *L. formosensis* may have been misidentified as *L. garvieae*.

The knowledge of virulence determinants in *L. garvieae* is limited, and even less is known about *L. petauri* and *L. formosensis* (18). Several studies have reported differences in the virulence gene content of *L. garvieae* isolates from human, fish, and food sources, as well as host-specific pathogenicity in animal models (18). The virulence gene content in *L. formosensis*, *L. garvieae,* and *L. petauri* has not been comparatively analyzed, and their population structures have not been comprehensively studied. In this study, we used WGS to analyze the population structure of *L. formosensis*, *L. garvieae,* and *L. petauri* and identify virulence genes associated with host specificity. The results are useful for improving our understanding of the infectious disease epidemiology of these organisms under the latest taxonomy.

## RESULTS

### Isolates included in the study

The genomes of 212 isolates were analyzed. These included eight *L*. *garvieae* isolates collected from patients with bacteremia in three hospitals in Hong Kong. In addition, 204 genome assemblies of *L. formosensis*, *L. garvieae,* and *L. petauri* from the National Center for Biotechnology Information (NCBI) were included. A detailed description of these 212 isolates is provided in Table S1 in File S1.

### Clinical and microbiological characteristics of the cases from Hong Kong

The details of the eight cases (L1 to L8) are summarized in Table 1. There were four male and four female patients. The median age was 78 years old (range, 58–92 years). All patients had multiple comorbidities. The most common presenting symptom was fever (four cases) followed by gastrointestinal symptoms (including abdominal pain, nausea, and vomiting, three cases). All patients had bacteremia with positive blood cultures. Two patients (L1, L4) had primary bacteremia with no identifiable source or focal infection. Two patients had infective spondylodiscitis involving the cervical (L2) and lumbar spines (L3). The remaining four cases had neutropenic fever (L5), urosepsis (L6), cerebral mycotic aneurysm (L7), and ischemic colitis (L8). Case 7 reported a history of sushi and sashimi consumption 6 days before presenting with sudden onset of severe headache. None of the other cases had recent history of seafood or sushi/sashimi consumption prior to symptom onset. All patients received β-lactam antibiotics including ampicillin, amoxicillin–clavulanate, ceftriaxone, penicillin, piperacillin–tazobactam, and meropenem. Three patients received vancomycin. All patients survived the infective episodes and were discharged.

**TABLE 1:** Clinical and microbiological details of cases with *L. petauri*, *L. formosensis*, *and L. garvieae* bacteremia, Hong Kong, 2017–2022^*b*^

Characteristic				Case				
	L1	L2	L3	L4	L5	L6	L7	L8
Sex/age	F/84y	M/75y	F/85y	F/92y	M/78y	M/58y	M/59y	F/78y
Symptom onset date	Oct-2017	Jul-2018	Apr-2019	Apr-2019	Oct-2019	Apr-2020	Apr-2021	Jun-2021
Presenting symptoms	Chest discomfort, dizziness	Fever	Fever, back pain	Fever, dizziness, malaise, vomiting	Fever	Reduced urine output	Severe headache, vomiting	Nausea, vomiting, abdominal pain, SOB
Underlying disease	DM, HT, AF, Parkinson’s disease	HBV carrier, HT, AF, CHF, gout	HT, ESRF on hemodialysis, IHD	DM, HT, cardiac pacemaker	HBV carrier, B-cell lymphoma	Metastatic CA sigmoid	CRHD	HT, AF, IHD, CA rectum in remission
Diagnosis	Primary bacteremia	Bacteremia, infective spondylodiscitis	Bacteremia, infective spondylodiscitis, bilateral psoas abscess	Primary bacteremia	Bacteremia, neutropenic fever	Bacteremia, urosepsis	Bacteremia, cerebral mycotic aneurysm	Bacteremia, ischemic colitis
Antibiotic treatment	AUG	VAN, CRO	AMP, PEN, AMC	AUG, CRO	MEM, CRO	AUG, CXM, TZP	CRO, VAN	TZP, MEM, VAN, CRO
Complications, outcome	Survived	Survived	Survived	Survived	Survived	Survived	Intracranial hemorrhage, survived	Survived
Identification								
MALDI-TOF	*L. garvieae*	*L. garvieae*	*L. garvieae*	*L. garvieae*	*L. garvieae*	*L. garvieae*	*L. garvieae*	*L. garvieae*
WGS	*L. petauri*	*L. garvieae*	*L. petauri*	*L. petauri*	*L. formosensis*	*L. petauri*	*L. garvieae*	*L. petauri*
Susceptibility^*a*^								
Ampicillin	S	S	S	S	S	S	S	S
Clindamycin	R (*IsaD*, *mdtA*)	R (*IsaD*, *mdtA*)	R (*IsaD*, *mdtA*)	R (*IsaD*, *mdtA*)	R (*IsaD*, *mdtA*)	R (*IsaD*, *mdtA*)	S (*IsaD*, *mdtA*)	R (*IsaD*, *mdtA*)
Levofloxacin	S	S	S	S	S	R (*gyrA* S83R, *parC* S80R)	S	S
Linezolid	S	S	S	S	S	S	S	S
Tetracycline	S	I	R (*tetS*)	R (*tetS*)	S	R (*tetS*)	S	S
PopPUNK	LPSC-13	LGSC-2	LPSC-45	LPSC-3	LFSC-22	LPSC-44	LGSC-2	LPSC-13
MLST	ST35	nST105	ST35	ST29	nST87	nST81	nST85	ST35

^*a*^ S, susceptible; I, intermediate; R, resistant. Resistance determinant is given inside brackets [ ].

^*b*^ AF, atrial fibrillation; AMP, ampicillin; AUG, amoxicillin–clavulanate; CA, carcinoma; CHF, congestive heart failure; CRHD, chronic rheumatic heart disease; CRO, ceftriaxone; DM, diabetes mellitus; ESBL, end-stage renal failure; F, female; HBV, hepatitis B virus; HT, hypertension; IHD, ischemic heart disease; M, male; MEM, meropenem; MLST, multilocus sequence typing; nST, new sequence type; PEN, penicillin; PopPUNK, Population Partitioning Using Nucleotide K-mers; R, resistant; S, susceptible; ST, sequence type; TZP, piperacillin–tazobactam; VAN, vancomycin; y, year.

The antimicrobial susceptibilities of the isolates are shown in Table 1. All isolates were susceptible to ampicillin, linezolid, and vancomycin. All isolates, with the exception of L7, are clindamycin resistant. Three isolates (L3, L4, L6) were resistant to tetracycline. Isolate L6 was resistant to levofloxacin.

The species identification of the eight isolates was obtained using three WGS-based methods. The Genome Taxonomy Database Toolkit (GTDB-Tk) identified five isolates as *L. petauri*, two as *L. garvieae,* and one as *L. formosensis* (Table 1). Identical species results were obtained using fast average nucleotide identity (ANI) and digital DNA–DNA hybridization (dDDH).

All eight isolates had two clindamycin resistance genes including *mdtA* (multidrug-resistance efflux protein) and *IsaD* (ribosomal protection protein). The tetracycline resistance gene, *tetS,* was detected in three isolates (L3, L4, L6). In isolate L6, an S83R substitution in the *gyrA* gene and an S80R substitution in the *parC* gene were detected. No quinolone resistance-determining region mutation was detected in the other seven isolates.

### Bacterial identification and analysis by PopPUNK and multilocus sequence typing (MLST)

The data set included 212 genomes. The collection sources include 92 from humans, 46 from bovines, 43 from rainbow trout, and 31 from other sources (File S1; Table S1). The 92 human isolates included 20 from human infections (16 from bacteremia and one each from cholecystitis, endocarditis, urinary tract infection, and finger wound infection) and 72 from human fecal samples. The geographic origin was available for the following 210 isolates: 131 from Asia, 59 from Europe, 19 from North America, and one from Africa.

The proportion of isolates with 100% and ≥90% genome completeness was 53% and 68% for *L. petauri*, 69% and 87% for *L. garvieae,* and 25% and 96% for *L. formosensis* (Table S1). The species assignments of the 212 isolates in the data set were obtained using GTDB-Tk, fast ANI, and dDDH. GTDB-Tk identified 120 as *L. petauri*, 53 as *L. formosensis,* and 39 as *L. garvieae* (File S1; Table S2). Identical species results were obtained using fast ANI and dDDH. A total of 77 isolates previously described as *L. garvieae* were re-classified as *L. formosensis* (*n* = 44) and *L. petauri* (*n* = 33) (Table 2).

**TABLE 2:** Summary of 77 isolates previously identified as *L. garvieae* that were re-identified as *L. formosensis* (*n* = 44) or *L. petauri* (*n* = 33) by WGS-based methods in the present study

Initial species identification and source^*a*^	Species identification by WGS-based methods
3 *L*. *garvieae* isolates (CP1, CP2, 1336) from lactococcosis outbreaks in rainbow trout, Spain, 1991–1996 (19)	All isolates were re-identified as *L. petauri*
*L. garvieae* strain 21881 isolated in a case of human bacteremia in 2007 (20, 21)	The strain was re-identified as *L. petauri*
*L. garvieae* strain 8831 isolated from rainbow trout lactococcosis outbreaks in Spain (21–23)	The strain was re-identified as *L. petauri*
*L. garvieae* strain I113 from meat sources, Italy, 2005 (24)	The strain was re-identified as *L. petauri*
*L. garvieae* strain M14 from Algerian fermented milk (25)	The strain was re-identified as *L. petauri*
8 *L*. *garvieae* isolates (four from human bacteremia, one from human wound, and three from fish), Singapore, 2012–2018 (26)	Four isolates were re-identified as *L. formosensis* and four as *L. petauri*
4 *L*. *garvieae* (*optrA* positive) isolates from stool samples of healthy adults, China, 2019 (27)	All isolates were re-identified as *L. petauri*
9 *L*. *garvieae* isolates from a fecal microbiota cohort (28)	All isolates were re-identified as *L. petauri*
3 *L*. *garvieae* blood culture isolates from three patients who experienced platelet concentrate-related bacteremia (29)	All isolates were re-identified as *L. petauri*
39 *L*. *garvieae* isolates from bovine mastitis, China, 2018–2021 (30)	38 isolates were re-identified as *L. formosensis* and one as *L. petauri*
*L. garvieae* strain TB25 from Italian cheese in 2001 (31)	The strain was re-identified as *L. petauri*
6 *L*. *garvieae* isolates including 2 from dairy products (FDAARGO_1062, KS1546), 2 from bovine sources (FDAARGOS_1063), and 1 each from human acute cholecystitis (Lg-ilsanpaik-gs201105) and yellowtail (strain 122061) (30, 32)	One (strain 122061) was re-identified as *L. formosensis*; the other five strains were re-identified as *L. petauri*

^*a*^ The collection sources of the re-classified isolates were as follows: 41 bovines, 10 human infection, 13 human fecal samples, five rainbow trout, and eight others (four fish, two milk, one pork sausage, and one cheese).

Among the 53 *L*. *formosensis* isolates, 42 gave higher dDDH values with type strain LMG 30663 (85.6%–100%) than with type strain NBRC 109475 (78.3%–82.2%) and were identified as *L. formosensis* subsp. *bovis*, whereas 11 isolates yielded higher dDDH values with NBRC 109475 (78.1%–100%) than LMG 30663 (73.8%–78.3%) and were identified as *L. formosensis* subsp. *formosensis* (File S1; Table S3). The same subspecies results were obtained by ANI value analysis. The 42 *L*. *formosensis* subsp. *bovis* isolates had ANI values of 97.5%–98.0% with NBRC 109475 and 98.2%–100% with LMG 30663. The 11 *L*. *formosensis* subsp. *formosensis* isolates had ANI values of 97.4%–100% with NBRC 109475 and 96.9%–97.5% with LMG 30663. The 42 *L*. *formosensis* subsp. *bovis* isolates included 38 from bovine mastitis and one each from bovine dump, human feces, swine, and pork sausage. The 11 *L*. *formosensis* subsp. *formosensis* included three from human infections (two from bacteremia and one from finger wound), three from human fecal sources, three from fish sources and one each from fermented broccoli and a beetle (*Zophobas atratus*) larva (Table S3).

**Fig 1:**
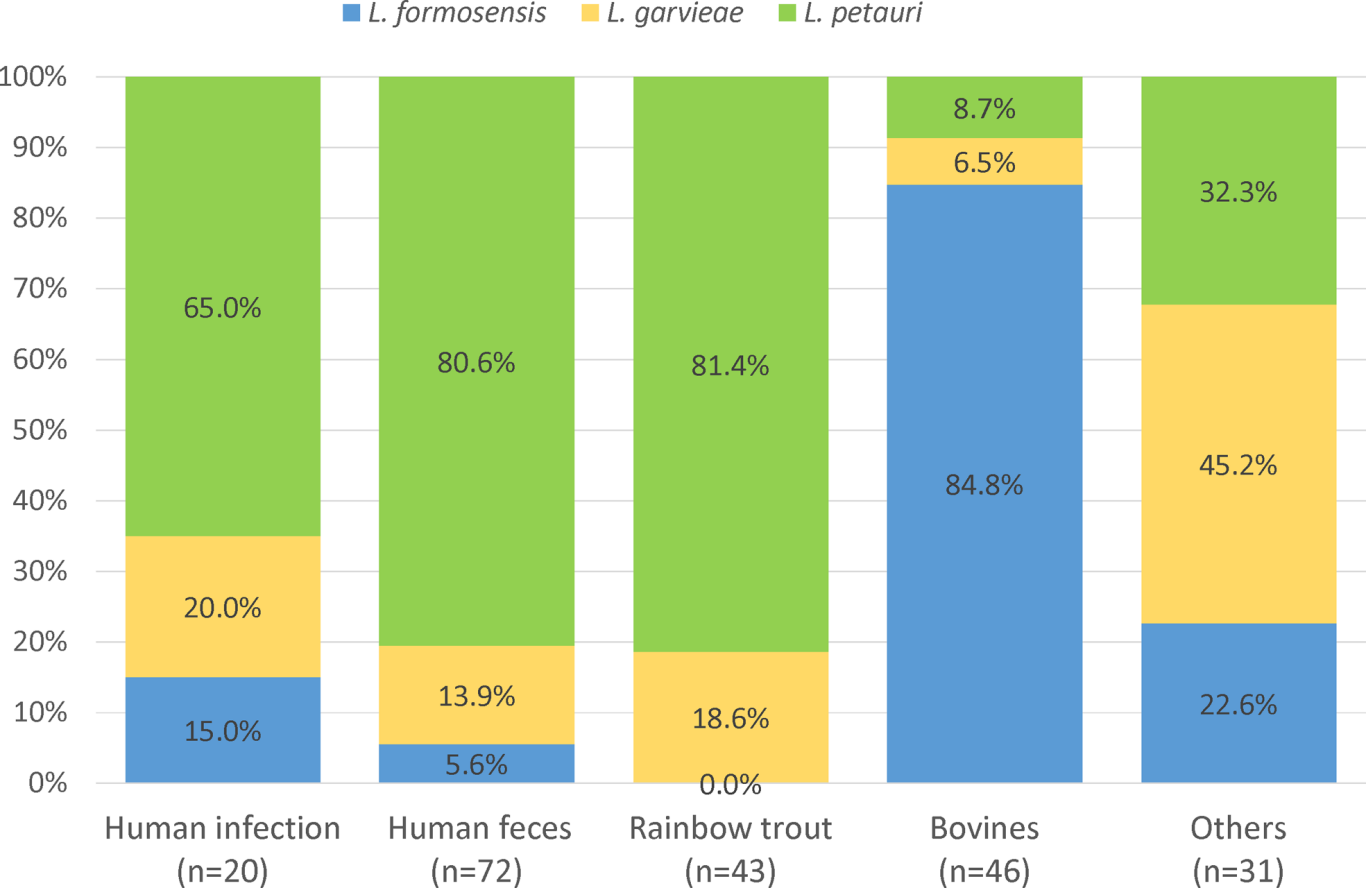
Distribution of *L. petauri*, *L. formosensis,* and *L. garvieae* by isolate sources.

Difference in the species distribution by collection sources were observed (Fig. 1). Among isolates from human infection, human feces, and rainbow trout sources, *L. petauri* is the predominant species comprising 65.0%, 80.6%, and 81.4% of the isolates, respectively. By comparison, *L. formosensis* comprised 84.8% of the isolates from bovines.

The genetic diversity of the isolates for the three species were high (Table 3). Population Partitioning Using Nucleotide K-mers (PopPUNK) produced more clusters than MLST types for *L. petauri* (45 vs 30) and *L. formosensis* (22 vs 18) (File S1; Table S4). Conversely, MLST produced more types than PopPUNK for *L. garvieae* (20 vs 13). Small differences in the Simpson’s diversity index for the two clustering methods in the three species were detected. The adjusted Rand index indicated that clustering agreement between the two methods was high for *L. formosensis* (0.949), moderately high for *L. petauri* (0.843) and relatively low for *L. garvieae* (0.524).

**TABLE 3:** Genetic diversity of *L. petauri*, *L. formosensis*, *and L. garvieae* by PopPUNK and MLST^*a*^

	*L. petauri*	*L. formosensis*	*L. garvieae*
No. of isolates	120	53	39
No. of PopPUNK clusters	45	22	13
No. of MLST types	30	18	18
Simpson’s diversity index			
PopPUNK	0.892 (0.849–0.935)	0.892 (0.842–0.941)	0.868 (0.804–0.931)
MLST	0.870 (0.829–0.910)	0.882 (0.834–0.929)	0.949 (0.920–0.978)
Adjusted Rand index	0.843	0.949	0.524

^*a*^ The adjusted Rand index indicates concordance between PopPUNK clusters and MLST types, where identical clustering is one, and completely different clustering is zero.

**Fig 2:**
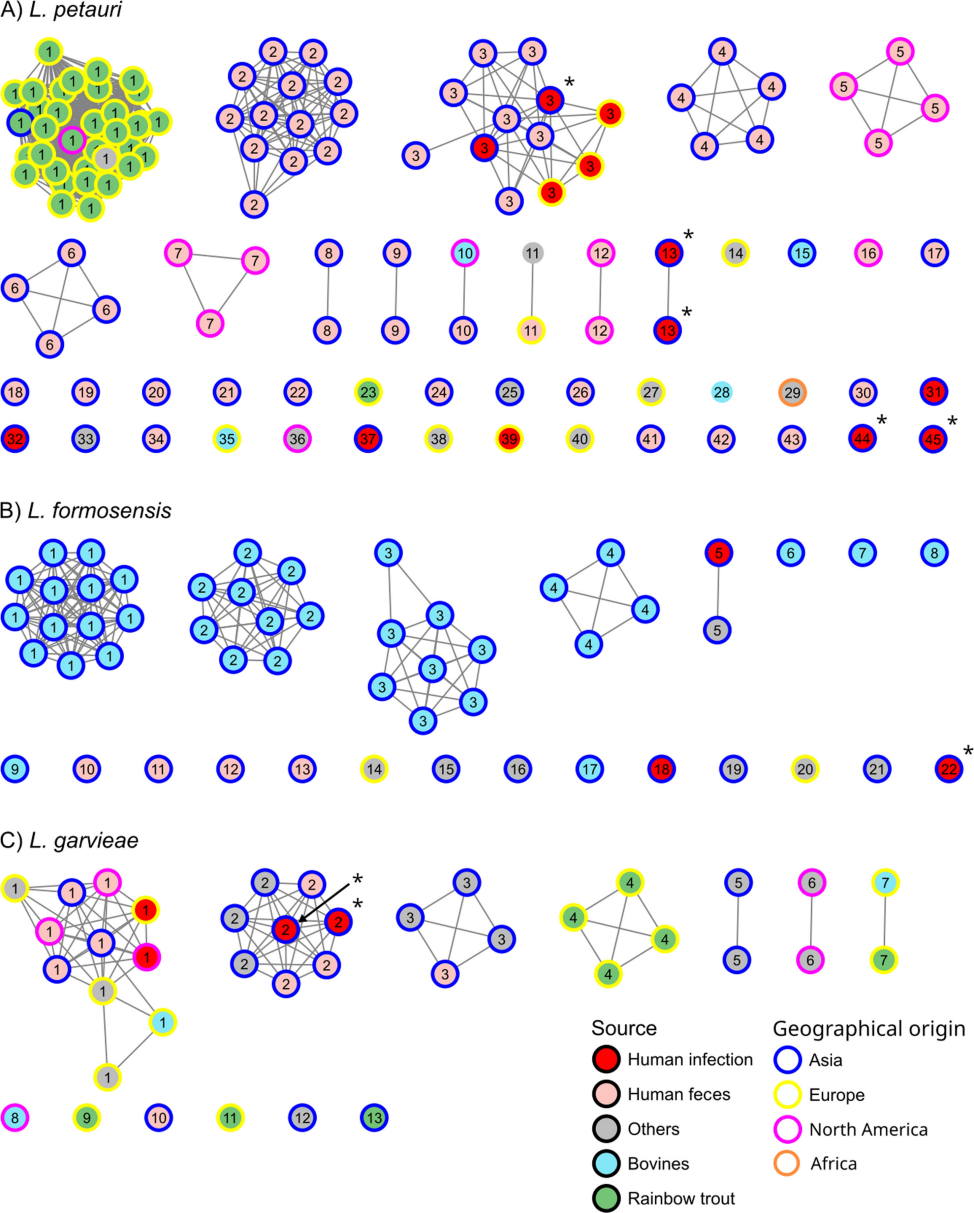
PopPUNK analysis *L. petauri*, *L. formosensis, and L. garvieae*. Network analysis for (**A**) 53 *L. formosensis*, (**B**) 39 *L*. *garvieae*, and (**C**) 120 *L*. *petauri* genomes labeled by their respective sequence cluster number. The nodes were colored by the isolate sources (inner fill) and geographical origin (outer circle). Asterisk indicates the eight isolates sequenced in this study. The *L. formosensis* subsp. *bovis* and *L. formosensis* subsp. *formosensis* isolates were partitioned into 12 (1 to 4, 6 to 9, 13, 15, 17, 20) and 10 clusters (5, 10–12, 14, 16, 18, 20, 26, 33), respectively.

The PopPUNK clusters of the isolates against collection source and geographical origin is shown in Fig. 2. The median number of single-nucleotide polymorphism (SNP) differences for between-cluster and within-cluster comparisons was 16,094 and 72 for *L. petauri*, 7,629 and 27 for *L. formosensis*, and 15,926 and 1,784 for *L. garvieae*, respectively (File S2; Fig. S1).

PopPUNK partitioned the 120 *L*. *petauri* isolates into 45 genomic clusters (LPSC-1 to -45) (Fig. 2A). Thirty-four *L. petauri* isolates from rainbow trout (20 from Spain, seven from Turkey, four from Greece, one each from Israel, Portugal, and USA during 1991–2020) and one isolate from a fermented fruit (Norway, 2017) were assigned to genomic cluster LPSC-1. The four bovine *L. petauri* isolates were assigned to genomic clusters with one isolate each. The 71 human *L. petauri* isolates were grouped into two major genomic clusters, LPSC-2 (*n* = 13, all from China) and LPSC-3 (*n* = 12, including seven from China, three from Italy, and one each from Hong Kong and Singapore), and 32 other genomic clusters with one to five isolates each. Two clusters contained isolates from human and food sources, including LPSC-10 (one from human fecal sample and one from ground beef with 872 SNP differences in pairwise comparison) and LPSC 11 (one from human fecal sample and one from dairy product with two SNP differences in pairwise comparison) (File S1; Table S5).

PopPUNK partitioned the 53 *L*. *formosensis* isolates into 22 genomic clusters (LFSC-1 to -22) (Fig. 2B). Four (LFSC-1 to -4) were larger clusters with four to 13 isolates each. The remaining five clusters (LFSC-6 to -9 and -17) had one isolate each. The seven *L*. *formosensis* human isolates were assigned to seven singleton clusters. The 39 bovine *L. formosensis* isolates (38 from China during 2018–2021 and one from India) were grouped into nine genomic clusters. Clusters containing the *L. formosensis* isolates from bovine sources did not contain any human isolates. One cluster (LFSC-5) contained two isolates from a human wound and a fish sample with 21 SNP difference in pairwise comparison (File S1; Table S6).

The 39 *L*. *garvieae* isolates were partitioned into 13 genomic clusters (LGSC-1 to -13) (Fig. 2C). These included four clusters (LGSC-1 to -4) with four to 11 isolates each and nine clusters with one to two isolates each. Thirteen of the 14 human *L. garvieae* isolates belong to three genomic clusters that also contained isolates from other non-human sources (such as cheese, fermented pitaya juice, turkey meat, tilapia, yellowtail). These included LGSC-1 (*n* = 11 including five from Europe, three from Asia, and three from North America during 1988–2019), LGSC-2 (*n* = 6, from China, Japan, and South Korea during 1974–2019), and LGSC-3 (*n* = 4, from China and Singapore during 2014–2015). The intra-cluster SNP difference among the isolates in LGSC-1, LGSC-2, and LGSC-3 was 102 to 8,540, 24 to 1,962, and 3 to 248, respectively (File S1; Table S7).

**Fig 3:**
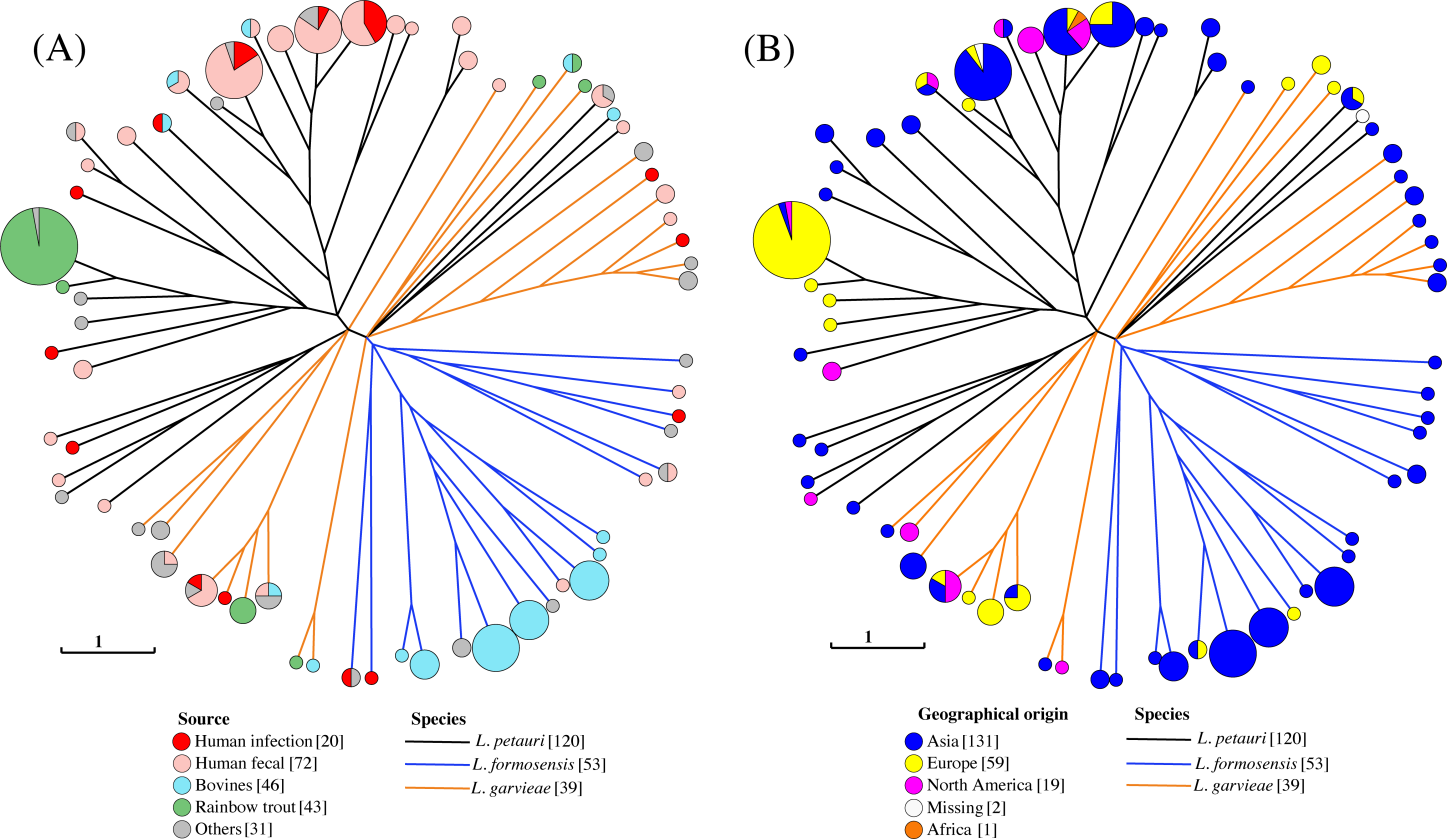
Visualization of MLST genetic clusters for *L. petauri*, *L. formosensis,* and *L garvieae*. Each node represents one MLST sequence type. The isolate source (**A**) and geographical origin (**B**) are indicated by colors in the nodes. The species are indicated by color branches. The scale bar indicates distance for one allele difference.

The isolates of *L. petauri*, *L. formonesis,* and *L. garvieae* were classified into 30, 18, and 20 MLST types, respectively (File S1; Table S4). The MLST types based on isolate source and geographical origin were similar to the clustering obtained by PopPUNK (Fig. 3). The number of MLST types with isolates from human and non-human sources for *L. petauri*, *L. formonesis,* and *L. garvieae* was 7, 2, and 3, respectively. However, the majority of human and non-human isolates with shared MLST types were resolved into distinct genomic clusters by PopPUNK (File S1; Table S8).

Out of our eight cases, the five *L*. *petauri* isolates were partitioned to four genomic clusters (LPSC-3, -13, -44, -45) and three MLST types (ST29, ST35, nST81). The two *L*. *garvieae* isolates belonged to LGSC-2 and -2 MLST types (nST85, nST105), while the only *L. formosensis* belonged to LFSC-22 and nST86 (Table 1). nST81, nST85, nST87, and nST105 are new MLST types detected in this work.

### Virulence gene profiles by species

To gain insight into the genetic features associated with virulence, we queried the presence and absence of virulence genes in all the isolates. In total, 68 virulence genes were detected in the 212 isolates (File S1; Table S9). These included 29 adherence genes, nine iron uptake genes, three hemolysin genes, seven enzyme-related virulence genes, 15 genes in the capsule gene (*cps*) cluster, and five genes in the biofilm enhancer in *Enterococcus* (*bee*) locus.

**Fig 4:**
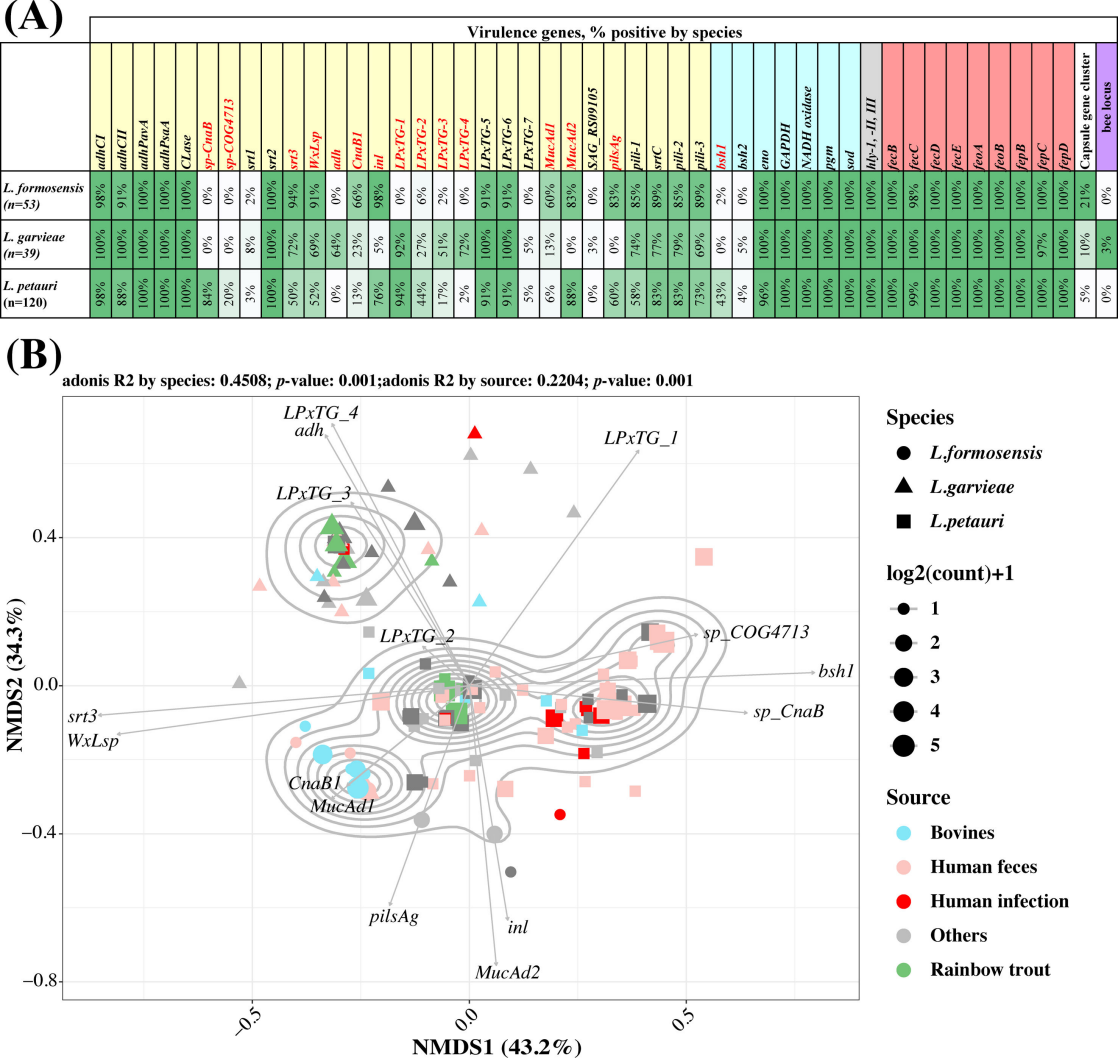
Virulence genes by species and source. (**A**) Heatmap showing presence of virulence genes in *L. petauri*, *L. formosensis*, and *L. garvieae*. Color of the gene labels on the top panel indicates the group of the virulence genes: adherence (yellow), enzyme-related virulence genes (light blue), hemolysin (gray), iron uptake (red), capsule gene cluster (white), and *bee* locus (purple). The 15 virulence genes, which presence were significantly different among the three species, were indicated in red font. (**B**) Non-metric multidimensional scaling (NMDS) analysis of virulence genes in *L. formosensis*, *L. garvieae*, and *L. petauri*. NMDS based on the presence and absence of 15 virulence genes (*sp-CnaB*, *sp-COG4713*, *srt3*, *WxLsp*, *adh*, *CnaB1*, *inl*, *LPxTG-1*, *LPxTG-2*, *LPxTG-3*, *LPxTG-4*, *MucAd1*, *MucAd2*, *pilsAg*, and *bsh1*). Symbols and colors are used to indicate the species and sample source of the isolate. The symbol size indicates the number of isolates. Contour lines, which are generated by two-dimension (2D) kernel density estimation, show the clustering of communities from 2D ordination space values. A biplot is used to indicate the contribution of the 15 virulence genes on the 2D axes. The difference in the distribution of the virulence genes by species (Adonis R^2^ 0.4508, *P* = 0.001) and by sample source (Adonis R^2^ 0.2204, *P* = 0.001) are both statistically significant.

Out of the 29 adherence genes, 12 (*adhCI*, *adhCII*, *adhPavA*, *adhPsaA*, *CLase*, *srt2*, *LPxTG-5*, *LPxTG-6*, *pili-1*, *srtC*, *pili-2*, and *pili-3*) were shared by a majority of the isolates across the three species. These genes were present in 85%–100% of *L. formosensis*, 69%–100% of *L. garvieae*, and 58%–100% of *L. petauri* (Fig. 4A). On the other hand, the presence of 14 adherence genes (*sp-CnaB*, *sp-COG4713*, *srt3*, *WxLsp*, *adh*, *CnaB1*, *inl*, *LPxTG-1*, *LPxTG-2*, *LPxTG-3*, *LPxTG-4*, *MucAd1*, *MucAd2*, and *pilsAg*) varied across the three species, with percentages ranging 0%–98% in *L. formosensis*, 0%–92% in *L. garvieae*, and 0%–94% in *L. petauri*. The presence of these genes differed statistically between species (Fisher exact tests, adjusted *P* < 0.05 for all 14 virulence genes). The remaining three adherence genes (*srt1*, *LPxTG-7*, and SAR_RS09105) were either absent or rare in all three species.

Out of the nine iron uptake genes, seven genes (*fecB*, *fecD*, *fecE*, *feoA*, *feoB*, *fepB*, and *fepD*) were present in all isolates, while two genes (*fecC* and *fepC*) were present in 97%–100% of the isolates by species (Fig. 4A). All isolates had the three hemolysin genes. Five enzyme-related virulence genes (*eno*, *GAPDH*, *NADH oxidase*, *pgm*, and *sod*) were detected in almost all isolates. Regarding bile salt hydrolase genes, *bsh1* was significantly more prevalent in *L. petauri* than in *L. formosensis* and *L. garvieae*, while *bsh2* was rare. (Fig. 4A).

The *cps* cluster genes were detected in 21 (10%) isolates, comprising 11 (21%) *L*. *formosensis* isolates, four (10%) *L*. *garvieae* isolates, and six (5%) *L*. *petauri* isolates. In the reference Lg2 genome, the *cps* cluster consisted of 15 genes. The number of *cps* genes varied among the isolates. Out of the 15 genes, four (*espA*, *espB*, *espC*, and *espD*) were present in all 21 isolates, while four genes (*epsR*, *epsX*, *ugdh*, and *cpsL*) were detected in 18–20 isolates. The remaining seven genes (*rtf*, *gtf*, *atf*, *gtff4p*, *wzy*, *wzx*, and *ugdh*) were found in two to three isolates. *L. formosensis* had four to eight *cps* genes, *L. garvieae* had eight to 15 *cps* genes, and *L. petauri* had seven to nine *cps* genes. Only two *L. garvieae* isolates (Lg2 and JJJN1) possessed the complete set of 15 *cps* genes in the entire data set.

The 15 virulence genes, comprising 14 adherence genes and *bsh1*, which showed significant differences among the three species, were subjected to non-metric multidimensional scaling (NMDS) analysis. The analysis revealed differential clustering by species (R^2^ 0.4508, *P* = 0.001) and by sample source (R^2^ 0.2204, *P* = 0.001) (Fig. 4B). The biplot showed that three genes (*sp_COG4713*, *bsh1*, and *sp_CnaB*) were associated with human *L. petauri*, two genes (*CnaB1* and *MucAd1*) were associated with *L. formosensis* from bovine and human sources, and three genes (*LPxTG-4*, *adh*, and *LPxTG-3*) were associated with *L. garvieae* from multiple sources.

## DISCUSSION

This study reveals that many human isolates previously identified as *L. garvieae* are actually *L. petauri*, including approximately two-thirds of our blood culture isolates. A similar proportion of the previously published *L. garvieae* human isolates were reclassified as *L. petauri* in this study (20, 26, 30). As *L. petauri* is a newly described species, there is only one case report of human urinary tract infection caused by this organism (33). The current study revealed earlier cases of human *L. petauri* infections in multiple countries from 2007 to 2020 (20, 26, 30). Retrospective reviews of case series have suggested that *L. garvieae* may have a propensity to cause infective endocarditis (5). However, those reports were published before the recognition of *L. petauri* as a distinct species, or the identification methods used were unable to distinguish between *L. petauri* and *L. garvieae*. Further research is needed to clarify the link between these species and endocarditis. The differential association of *L. petauri* with human host is further supported by the observation that an even higher proportion of human fecal isolates were *L. petauri*. In the PopPUNK analysis, the relatedness of isolates from human fecal and human infection sources is indicated by their co-occurrence in one large genomic cluster. In contrast, human *L. petauri* isolates were partitioned to genomic clusters that were distinct from those that caused lactococcosis in rainbow trout or those from other food sources. None of our five cases of *L. petauri* bacteremia had a recent history of consuming seafood, sushi, or sashimi before experiencing symptoms. This finding suggests that *L. petauri* infections in humans may more commonly arise from preexisting gut colonization rather than recent exposure to contaminated food sources.

The present study is the first to report human infections caused by *L. formosensis*, including one case sequenced in this study and two previously reported *L. garvieae* cases that were reclassified as *L. formosensis* (26). The preference for specific hosts among the two subspecies of *L. formosensis* is indicated by the observation that all isolates from bovine mastitis were *L. formosensis* subsp. *bovis*, while human-associated isolates, including our case L5, were predominantly *L. formosensis* subsp. *formosensis*. None of the isolates linked to human infections were classified as *L. formosensis* subsp. *bovis*. In contrast, all but one of the *L. garvieae* isolates previously described to cause mastitis in multiple farms in China from 2017 to 2021 were reclassified as *L. formosensis* subsp. *bovis* (30). The type strain of *L. formosensis* subsp. *bovis*, previously known as *L. garvieae* subsp. *bovis*, was first isolated from a gaur dung sample in India (34). In 2021, *L. garvieae* subsp. *bovis* was transferred to *L. formosensis* as *L. formosensis* subsp. *bovis* (35). Phenotypically, the inability to grow in brain heart infusion medium at pH 5, on tryptic soy agar with 4% NaCl and on De Man–Rogosa–Sharpe (MRS) agar at 42°C distinguished *L. formosensis* subsp. *bovis* from *L. formosensis* subsp. *formosensis* (34, 35). Nonetheless, the differential ability of the two subspecies to cause disease in different hosts has not been previously investigated.

Difference in virulence gene composition was observed both by species and by collection source. This could explain the lack of virulence of some human isolates on fish and why some isolates pathogenic to fish were not pathogenic in mice (36, 37). Additionally, it was reported that *L. garvieae* strains from terrestrial plants were nonpathogenic toward yellowtail and mice (38). It is important to acknowledge that previous studies reporting differences in virulence gene content or pathogenicity among individual *L. garvieae* strains are affected by misclassification of species. For example, two out of five *L. garvieae* isolates included in a genomic analysis were actually *L. petauri* (21), and in another study, 11 out of 24 *L*. *garvieae* genomes were in fact *L. petauri*, *L. formosensis*, or other species (32). Additionally, *L. garvieae* 8831, previously demonstrated to be able to invade non-phagocytic cells in rainbow trout, was reclassified as *L. petauri* in this study (22).

In Japan, there was an outbreak of lactococcosis in culture-stripped jack (*Pseudocaranx dentex*) in 2021 (39). Investigation of three representative strains (L21-12, L21-68, and L21-20) showed that the outbreak involved both *L. garvieae* and *L. formosensis* of different pathogenicity in experimental fish models (39). To place the three recent strains into the context of this study, we queried the genomes of the three isolates against our PopPUNK database. Strains L21-12 (GCA_037076365.1), L21-68 (GCA_037076375.1), and L21-20 (GCA_037101245.1) were classified as LGSC-2, LGSC-5, and LFSC-19, respectively. Interestingly, all the isolates in the three PopPUNK clusters in our database were collected from Asia (Fig. 2). These include eight LGSC-2 isolates (two from diseased yellowtail in Japan, one from So-iuy mullet in South Korea, and five from humans in mainland China and Hong Kong), two LGSC-5 isolates (one from greater amberjack in Japan and one from yellow croaker in China), and one LFSC-19 (from diseased yellowtail in Japan).

This study has revealed the association between three virulence genes (*sp_COG4713*, *bsh1*, and *sp_CnaB*) and human *L. petauri* isolates. It has been reported that the two adherence genes (sp_COG4713 and sp_CnaB) are present in the human-pathogenic strain HF but absent in the fish-pathogenic strain 074 (37). The gene *bsh1*, which encodes a bile salt hydrolase, may contribute to human host adaptation by enhancing bacterial survival in the human gut. It has been reported that the gene *bsh1* is present in human and food *L. garvieae* isolates but not in *L. garvieae* isolates from rainbow trout (31). In the same study, it was found that exposure to bile salts upregulated the expression of *bsh1* and other virulence genes that encode adherence proteins and hemolysin (31).

Nonetheless, there are many shared genes among the pangenomes of *L. garvieae* and related species (32). This explains the presence of many virulence genes shared among *L. petauri*, *L. formosensis*, and *L. garvieae*, and the potential for all three species to cause disease in different hosts. Adhesins, LPxTGs, and sortases are interacting virulence factors that facilitate bacterial adhesion to tissues, host cell invasion, and evasion of the immune system (21, 22). A set of iron uptake genes, hemolysin genes, and enzyme-related genes are shared by many isolates across the three species, and these core genes contribute to a pathogen’s ability to invade, multiply, and cause damage in the infected host (40). The prevalence of the capsule gene cluster was low among the three *Lactococcus* species. It has been observed that the presence of capsule is not essential for the virulence of *L. garvieae* in fish, as many lactococcosis outbreaks were caused by noncapsulated strains (2, 41).

This study has several strengths, including a large data set, inclusion of high-quality genomes, and identification to species and subspecies level using the latest taxonomy. Furthermore, the isolates were partitioned using PopPUNK, which is more discriminatory than MLST. The analysis of virulence genes was performed using NMDS, a robust method that allows for statistical testing of group differences and visualization of relationships. However, this study has several limitations. First, the small number of isolates from sushi and sashimi precludes any meaningful analysis of this particular source. Second, the investigation focused only on the presence and absence of virulence genes, without considering their sequence variation. Finally, the study did not investigate the expression of virulence genes.

### Conclusion

This work provides epidemiological and genomic evidence of host specificity among the three species and also reveals species- and source-related differences in virulence gene composition. These results broaden our understanding of *L. petauri*, *L. formosensis*, and *L. garvieae* as pathogens in different hosts.

## MATERIALS AND METHODS

### Bacterial isolates, identification and antimicrobial susceptibility

This is a retrospective study on cases of *L. garvieae* bacteremia in three public, regional hospitals (KWH, PYH, and QMH) in Hong Kong, from 2017 to 2022. Cases of *L. garvieae* were retrospectively identified using the Laboratory Information System. The clinical information, including patient demographics, comorbidities, presenting symptoms, laboratory test results, antibiotic treatment, and outcomes, was retrieved from the electronic patient record of the Hospital Authority, which manages all public hospitals in Hong Kong. The isolates stored in Microbank (Pro Lab Diagnostics Inc., Toronto, ON, Canada) were retrieved and subcultured onto blood agar for testing. Bacterial identification was performed using MALDI-TOF MS (Bruker Daltonics, Bremen, Germany)

Antimicrobial susceptibility testing was performed using the disc diffusion method on blood Muller–Hinton agar according to the guidelines from the Clinical and Laboratory Standards Institute (42). Since there is no organism-specific, susceptibility breakpoints for *L. garvieae*, inhibition zone diameters (mm) from *Enterococcus* spp. and *Streptococcus* spp. viridans groups were used to interpret the disc results [sensitive (S), intermediate (I), resistant (R)]: 10 µg of ampicillin (S, ≥17; R, ≤16), 2 µg of clindamycin (S, ≥19; I, 16–18, R, ≤15), 5 µg of levofloxacin (S, ≥17, I, 14–16, R, ≤13), 30 µg of linezolid (S, ≥21), 30 µg of tetracycline (S, ≥19; I, 15–18; R ≤ 14), 30 µg of vancomycin (S, ≥17) (42).

### DNA extraction, sequencing and quality control

In the present study, protocol for genomic DNA processing and analyses were as per our previous publications (43–45). Genomic DNA was extracted from cultures using QIAsymphony (Qiagen, Hilden, Germany) and then qualified with a Qubit fluorometer (Invitrogen, CA, United States). Sequencing was conducted at the Novogene Bioinformatics Institute (Beijing, China) with an Illumina NovaSeq 6000 or iSeq100 platform (Illunina, CA, United States). Long-read sequencing was also performed for all eight genomes using Nanopore MinION sequencer in our laboratory (44). All reads were filtered using Trimmomatic v0.39 or NanoFilt v2.8.0 as we did before (44).

### Genome assembly, annotation, and genome data set curation

Hybrid assemblies were performed for qualified reads using Unicycler v0.50 and evaluated using QUAST v5.0.2 after running an improvement pipeline from Sanger Institute as we did in a previous study (44). To further explore the phylogenetic relationship of our eight isolates with published ones, they were integrated with a global data set of 204 genomes (including 115 *L*. *petauri*, 52 *L*. *formosensis*, and 37 *L*. *garvieae*), which were retrieved from NCBI and previous publications (accessed on 25 October 2023) (Table S1). Information on biosamples of these reference genomes was downloaded from GenBank or retrieved from relevant publications. To make them comparable with our own genomes, all genomes used in the present study were qualified and annotated with the same pipeline. The quality of genomes was evaluated using CheckM v1.2.2 and annotated using Prokka v1.14.5 with a database curated for the genus *Lactococcus* from all annotated genomes deposited at GenBank (46, 47). High-quality draft genomes with CheckM completeness values in the range of substantially completed (≥70% to 90%) and near complete (≥90%), and low contamination (≤5%) were included (46). Genomes not meeting these completeness and contamination criteria were filtered out.

### WGS-based species identification

The taxonomy of all genomes was determined using three methods. The Genome Taxonomy Database Toolkit (GTDB-Tk v2.3.2) with database release 214 was performed to classify all genomes (48). The ANI of each genome was calculated for each genome with genomes of type strains, including *L. formosensis* subsp. *bovis* LMG 30663 (GCF_018403725.1), *L. formosensis* subsp. *formosensis* NBRC 109475 (GCF_018403745.1), *L. garvieae* ATCC 49156 (GCF_000269925.1), *L. garvieae* FDARGOS_929 (GCF_016026695.1), and *L. petauri* 159469 (GCF_002154895.1) using FastANI (49). The Type (Strain) Genome Server was used to calculate the dDDH of genomes from the present study and closely related type strains (50). ANI (≥95%–96%) and dDDH (≥70%) values were adopted as thresholds for species demarcation (49, 50).

### Antibiotic resistance genes (ARGs) and chromosomal resistance mutations

ARGs were identified following the protocol used in a previous study (44). Resistance Gene Identifier v6.0.3 was run against CARD databases 2023 release v3.2.8 with –include_nudge (51). Chromosomal resistance gene mutations were further clarified through aligning with reference genes, including topoisomerase-encoding genes *gyrA* (WP_004258266.1), *gyrB* (WP_002283665.1), and *parC* (WP_042218931.1).

### Virulence factors

Virulence factors were predicted using blastp on all deduced protein sequences against the Virulence Factor Database (VFDB) 2022 (52) and a customized *L. garvieae* virulence gene database, which was curated referring to literature (2, 30, 37, 41). All hits were filtered with 80% identity and 70% coverage as cutoffs and parsed into categories designated by VFDB or the relevant publications. The presence of virulence genes in *L. petauri*, *L. formosensis*, and *L. garvieae* were compared using Fisher exact tests. The panel of virulence genes that was significantly different among *L. petauri*, *L. formosensis*, and *L. garvieae* was further analyzed using NMDS, which was performed with the Bray–Curtis distance matrix calculated from a presence–absence matrix using Vegan R package (https://github.com/vegandevs/vegan). A permutational multivariate analysis of variance (Adonis) test for two variables (species and isolation source) was conducted with 999 permutations. The influence of variables on virulence gene composition was determined via the envfit function of Vegan, which is depicted as vectors. The length of the vectors is proportional to the degree of influence of the respective variables. Since some genomes overlapped, the counts of overlapped genomes were scaled in size of ordination points. The plot was generated using the ggplot2 package (https://ggplot2.tidyverse.org/).

### Phylogenomic analysis and multilocus sequence typing

Phylogenomic analysis was performed as in our previous work using ParSNP v1.1.2 to call core genome and harvest SNPs from genome alignment (44). The phylogenetic tree was constructed using IQ-TREE v2.2.0 with the best model proposed from ModelFinder and visualized using iTOL (https://itol.embl.de) (53). PopPUNK v2.6.0 was employed to cluster all 212 genomes, and the network of clusters was generated using Cytoscape v3.9.0 (54). MLST profiles of all genomes were determined via mlst (https://github.com/tseemann/mlst) with updated profiles from PubMLST (https://pubmlst.org/) and subjected to GrapeTree for genetic relationships (55). New alleles or sequence types detected in this work were indicated using a prefix n before the allele and MLST type number. The Simpson’s index of diversity (95% CI) for the two clustering methods, PopPUNK and MLST, was calculated as described (56). The adjusted Rand index was used to assess concordance between MLST and PopPUNK (57). Both indices were calculated using an online tool (http://www.comparingpartitions.info/) developed by the Institute of Microbiology, Faculty of Medicine, University of Lisbon, Portugal.

### Statistical methods

Fisher’s exact test was used to compare proportions. *P* values for the virulence gene comparisons were adjusted for multiple tests using the Bonferroni correction (58). An adjusted *P* value of <0.05 was considered to indicate statistical significance. All analyses were performed using R statistical software (version R-4.3.2).

## Data Availability

The sequences of the isolates in this study were deposited in the GenBank database under Bioproject PRJNA1074855.

## SUPPLEMENTAL MATERIAL

Figure S1 spectrum.00541-24-s0001.pdf Violin plots of the distribution of between-cluster and within-cluster SNP distances.

Supplemental tables spectrum.00541-24-s0002.xlsx Tables S1-S9.

ASM does not own the copyrights to Supplemental Material that may be linked to, or accessed through, an article. The authors have granted ASM a non-exclusive, world-wide license to publish the Supplemental Material files. Please contact the corresponding author directly for reuse.
