# Supplementary material for: Genomic investigation of Lactococcus formosensis, Lactococcus garvieae, and Lactococcus petauri reveals differences in species distribution by human and animal sources
Source: Microbiol Spectr. 2024 Apr 30;12(6):e00541-24. doi: 10.1128/spectrum.00541-24 (PMC11237765; doi:10.1128/spectrum.00541-24)

Figure S1. Violin plots of the distribution of between-cluster and within-cluster SNP distances for *L. petauri*, *L. formosensis* and *L. garvieae*.

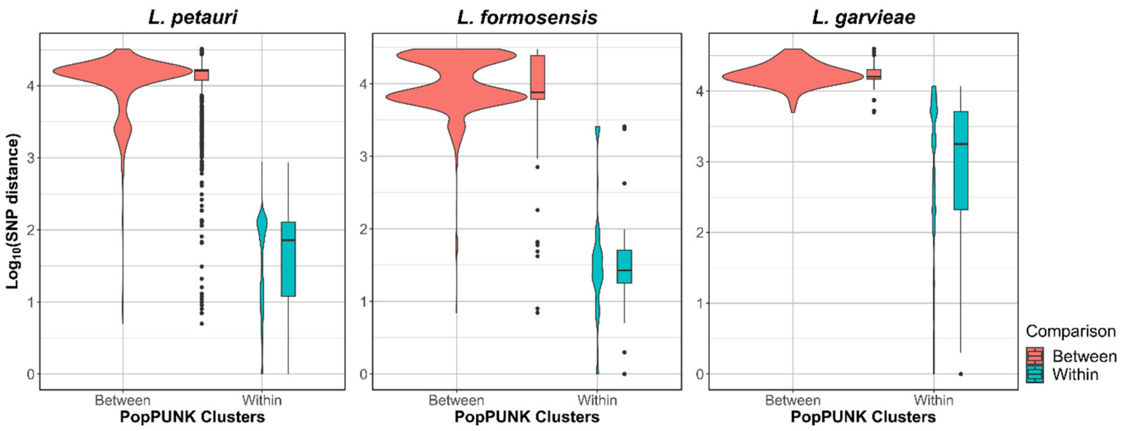

Supplement: Figure S1 — Violin plots of the distribution of between-cluster and within-cluster SNP distances. [file spectrum.00541-24-s0001.pdf]
